# An Exploratory Study to Test the Impact on Three “Bolt-On” Items to the EQ-5D

**DOI:** 10.1016/j.jval.2014.09.004

**Published:** 2015-01

**Authors:** Yaling Yang, Donna Rowen, John Brazier, Aki Tsuchiya, Tracey Young, Louise Longworth

**Affiliations:** 1 Nuffield Department of Primary Care Health Sciences, University of Oxford, Oxford, UK; 2 Health Economics and Decision Science, School of Health and Related Research, University of Sheffield, Sheffield, UK; 3 Department of Economics, University of Sheffield, Sheffield, UK; 4 Health Economics Research Group, Brunel University, Uxbridge, UK

**Keywords:** bolt-on, EQ-5D, health state valuation, hearing, tiredness, vision

## Abstract

**Background:**

Generic preference-based measures were criticized for being inappropriate in some conditions. One solution is to include “bolt-on” dimensions describing additional specific health problems.

**Objectives:**

This study aimed to develop bolt-on dimensions to the EuroQol five-dimensional questionnaire (EQ-5D) and assess their impact on health state values.

**Methods:**

Bolt-on dimensions were developed for vision problems, hearing problems, and tiredness. Each bolt-on dimension had three severity levels to match the EQ-5D. Three “core” EQ-5D states across a range of severity were selected, and each level of a bolt-on item was added, resulting in nine states in each condition. Health states with and without the bolt-on dimensions were valued by 300 members of the UK general public using time trade-off in face-to-face interviews, and mean health state values were compared using *t* tests. Regression analysis examined the impact of the bolt-on variants and the level of the bolt-on items after controlling for sociodemographic characteristics.

**Results:**

Bolt-on dimensions had an impact on health state values of the EQ-5D; however, the size, direction, and significance of the impact depend on the severity of the core EQ-5D state and of the bolt-on dimension. Regression analysis demonstrated that after controlling for possible differences in sociodemographic characteristics between the groups, there were no significant differences in health state values between the three bolt-on dimensions but confirmed that the impact depended on the severity of the EQ-5D health state and the levels of bolt-on dimensions.

**Conclusions:**

The impact of a bolt-on dimension on the EQ-5D depends on the core health state and the level of the bolt-on dimension. Further research in this area is encouraged.

## Introduction

Generic preference-based measures of health-related quality of life (HRQOL) are commonly used for evaluating the impact of health conditions and their treatments. The advantages of these measures include an ability to capture the impact of conditions or treatment on the overall HRQOL rather than focusing on specific symptoms and an ability to facilitate comparisons across different conditions and disease areas. Furthermore, the “preference-based” aspect of the measures enables the value people place on different health states or aspects of health to be reflected. Consequently, they are widely used for estimating quality-adjusted life-years and for capturing quality-of-life effects in economic evaluations.

The advantages of generic preference-based measures could, however, come at a price. Specifically, they may not capture all important health effects for all conditions and treatments, and therefore there may be circumstances in which these generic measures of HRQOL are not appropriate for assessing health benefit. Generic measures, including the EuroQol five-dimensional questionnaire (EQ-5D), have been criticized for being insensitive or failing to capture important aspects of health [1], [2]. When this arises, it leads to the challenge of how best to obtain health state preference data, particularly if there is a need to estimate quality-adjusted life-years. One possible solution is the development of new dimensions to “bolt-on” to existing generic preference-based measures.

The EQ-5D is a preference-based HRQOL instrument that has been used to measure health status for a wide range of conditions and diseases. Its descriptive system contains five dimensions: mobility, self-care, usual activities, pain/discomfort, and anxiety/depression. Each dimension has three levels of severity and can describe a total of 243 health states [3]. It is commonly used in economic evaluation and to inform health care decision making by organizations such as the National Institute for Health and Care Excellence (NICE) in the United Kingdom [4], [5]. From the outset of the development of the EQ-5D, it was recognized that it could not be “simple” and “comprehensive” at the same time [3]. Since then, the EQ-5D has been validated in a wide range of conditions. It may still not be appropriate, however, for all conditions, and recent reviews found that its performance in some specific disorders is poor [6], [7], [8].

There are two possible explanations for the failure of generic preference-based measures in some conditions. The first is that the range or number of descriptions of levels on each dimension of health is not sufficient to capture small changes within that area of health. The second is that descriptive systems may exclude an important dimension of health. The first problem of having too few levels to capture small changes in health may be overcome by increasing the size of the sample in which the data are obtained or increasing the number of levels of the instrument. This latter approach has been taken by the EuroQol Group through the publication of a five-level version of the EQ-5D [9]. The second issue is more problematic, but a potential solution is to bolt on additional item(s) to capture additional elements of HRQOL. The development of these bolt-on item(s) to the EQ-5D could enable researchers to retain the EQ-5D descriptive system as core and select additional dimensions to improve the content validity of the instrument for a particular condition. In the context of economic evaluation, the question of whether a bolt-on dimension is useful will depend on the extent to which values of EQ-5D health states are affected by the inclusion of the dimension. If the bolt-on dimension does not affect values, it would demonstrate that the impairment described by the dimension has little or no impact on health-related utility or that it is already captured by the five EQ-5D dimensions.

Previous studies have sought to investigate the addition of extra dimensions to the EQ-5D, including a cognition dimension [10] and sleep [11]. In addition, early work of the EuroQol Group examined the EQ-5D with an energy/tiredness dimension added on [12]. The added cognition dimension showed a significant impact on health state values of EQ-5D states, whereas the energy/tiredness and sleep dimensions did not.

The aim of this exploratory study was to test the impact of adding three potential bolt-on items to the EQ-5D and to quantify the effect each has on EQ-5D health state values. The three clinical areas addressed by the bolt-on items were identified as part of a larger Medical Research Council-National Institute for Health Research–funded project to examine the use of generic and condition-specific measures in NICE decision making. A series of systematic reviews to examine the validity and responsiveness of generic measures of HRQOL found that the performance of the EQ-5D was poor in hearing-related conditions [13] and in some specific vision disorders [6]. Therefore, hearing and vision disorders were selected as bolt-on dimension candidates for further consideration. A third area of “tiredness” was also selected because concerns about the ability of the EQ-5D to reflect energy, particularly cancer-related fatigue, has been highlighted in a recent review of how NICE measures the value of health care interventions [14].

## Methods

The overall study design involved allocating a representative general population sample into four groups, each valuing a set of EQ-5D states, with three groups valuing states with one of the bolt-on dimensions (vision, hearing, or tiredness) and the fourth group valuing EQ-5D states without bolt-on. This allowed a series of comparisons and regression analyses to be performed to estimate the effect of bolt-on variants and the levels they take.

### Development of the Three Bolt-On Items

Each dimension of health in the EQ-5D has a heading (mobility, self-care, usual activities, pain/discomfort, anxiety/depression), and the usual activities dimension has a clarification in parentheses: “Usual activities (e.g. work, study, housework, family or leisure activities).” Based on a brief review of existing quality-of- life and health status measures related to hearing, vision, and tiredness and the opinions of the research team, each bolt-on dimension was given a heading subtitle (hearing, vision, or tiredness). In addition, the bolt-on questions related to vision and hearing included clarifications in parentheses. This referred to glasses or contact lenses in the vision bolt-on—“Vision (using glasses or contact lenses if needed)”—and to hearing aids as an example in the hearing bolt-on—“Hearing (using equipment if needed, e.g. hearing aids).”

**Fig. 1:**
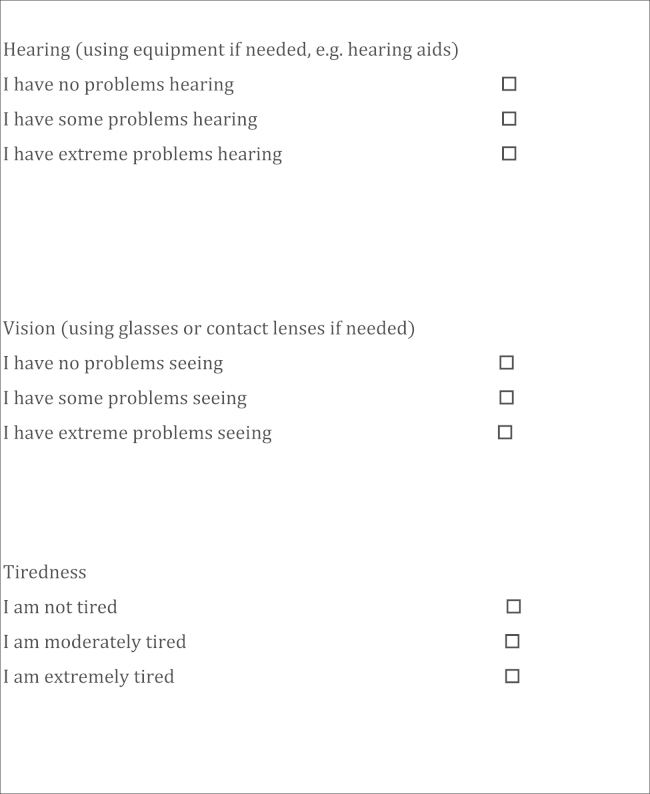
The three bolt-on items.

The description of severity levels of the bolt-on items follows the approach used for the three-level EQ-5D. The description of bolt-on items is presented in Figure 1.

### Selection of Health States for Valuation

**Fig. 2:**
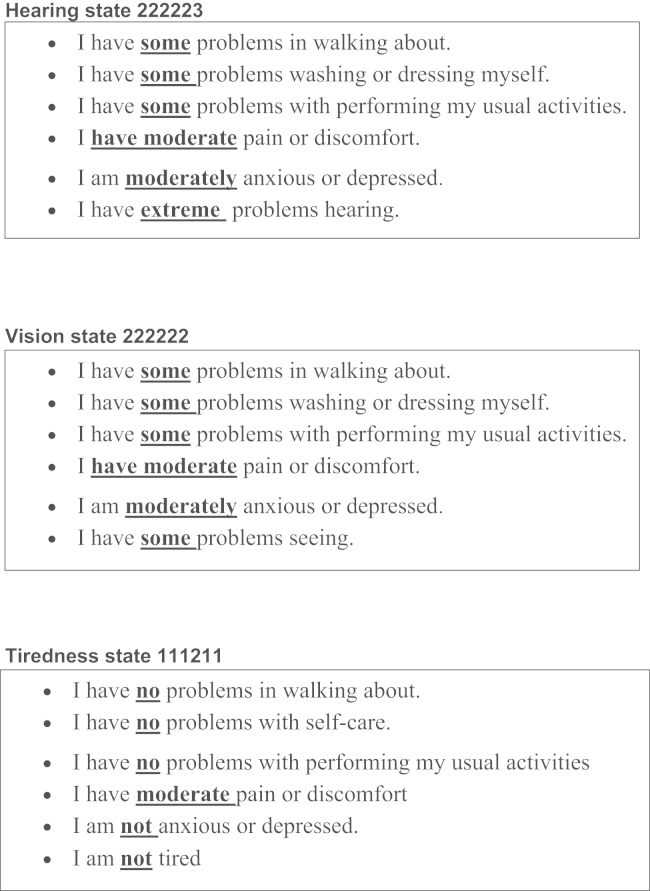
Examples of bolt-on health states valued.

Three EQ-5D health states were chosen as “core” states for valuation. The health states were selected after consideration of three criteria: 1) to cover a range of severity levels; 2) to select from the set of 43 states that have previously been valued in the Measurement and Valuation of Health (MVH) study, which was used to generate the social tariff of EQ-5D values for the United Kingdom [15], [16]; and 3) to include combinations of problems that are not implausible or rare. This third criterion was assessed by examining health states that occur with a relatively high frequency in the Health Survey for England [17]. The final selection included a “mild” state (11121), a “moderate” state (22222), and a “severe” state (22233). The classification of mild, moderate, and severe was based on observed utility values resulting from the MVH study, and the three states have a logically determined ordering of severity (mean value for 11121 = 0.85; mean value for 22222 = 0.50; and mean value for 22233 = −0.142). To each of the three core states three levels of the bolt-on item (with severity levels of 1, 2, or 3) were added, resulting in nine new states for each variant of the bolt-on instrument. These were given to three groups of respondents. The three core EQ-5D states without the bolt-on items were valued by a fourth group, and to increase the number of EQ-5D states from three to nine in line with bolt-on instruments, six more states were selected from the health states used in the previous large UK valuation study. Examples of bolt-on health states valued are shown in Figure 2.

### The Interviews

Assuming a power of 0.8, a significance level of 0.05, and an SD of 0.3 on the basis of a previous study [11], a sample size of 73 was required in each group to detect a difference of 0.1. To obtain 75 interviews per variant of the questionnaire, a valuation survey was undertaken using a sample of 300 people. Recruitment aimed to achieve a good spread across age, sex, ethnicity, and social class. The sample was selected on the basis of postal address within South Yorkshire using the Names and Numbers software [18]. Information sheets describing the project were sent to all households in the selected streets. Interviewers attended randomly selected households from those streets to obtain the residents’ consent to participate and conduct the interviews.

Individuals were randomly allocated into four groups: three groups each valued one of the four EQ-5D variants (three with a bolt-on dimension and one without). To minimize any interviewer effect, each interviewer undertook valuations of each questionnaire variant in turn. The interviews followed a format similar to that of the UK EQ-5D valuation study [15]. After agreeing to participate in the study, respondents for the three groups that included the bolt-on states were first asked to describe their own health using the EQ-5D and the bolt-on dimension they were about to value (as described in Fig. 1). Then, the respondents rated their own health using a visual analogue scale (VAS), in which the VAS was bounded by 0 (“worst imaginable health state”) and 100 (“best imaginable health state”). Then, respondents ranked six hypothetical states described on separate cards as a “warm-up” task. The cards described the EQ-5D health states or EQ-5D health states with a bolt-on dimension (depending on the allocated group) without the additional information in parentheses for the bolt-ons and usual activities dimensions. Note that the bolt-ons were first presented to respondents as part of self-rated health and asking them to report their levels of problems on these dimensions taking into account their use of aids (glasses, hearing aids, etc.). Short versions of the health states, which did not repeat this additional information, were then presented to respondents for valuation. These six consisted of four states randomly selected by interviewers or respondents from the nine states for each instrument, plus the best state described by the instrument and immediate death. The main objective of this stage was to familiarize respondents with the health state cards and stating their preferences toward the health states.

The main valuation exercise was conducted using the time trade-off (TTO) method [19]. The best health state (11111 or 111111) described by the given instrument was used as the upper anchor and “immediate death” was used as the lower anchor. The respondent was asked to imagine 10 years of life in the health state under valuation, relative to a shorter duration in the best state, both followed by immediate death. A “TTO board” was used as a visual aid to assist respondents, with one side for valuing health states better than dead and the other side for those health states worse than dead. To familiarize themselves with the TTO task, respondents were asked to complete an additional practice TTO task. Each respondent valued nine health states.

Finally, respondents were asked to complete sociodemographic questions and their health status according to the remaining bolt-on dimensions. For example, respondents valuing the hearing bolt-on dimension reported their hearing status at the start of the interview alongside the EQ-5D descriptive system, and then self-reported their vision and tiredness status after the valuation task as part of the background questions.

Five trained and experienced interviewers undertook the interviews. The project was approved by the Research Ethics Committee at Brunel University.

### The Analysis

The sociodemographic characteristics of the four groups of respondents were compared. A chi-square test was performed to examine whether there was any difference between the four samples in terms of categorical variables such as age, sex, or self-reported health status. For ordered variables (e.g., age groups and self-reported EQ-5D dimensions), the chi-square gamma statistic was undertaken to make adequate use of the relevant information. An analysis of variance test was performed to examine whether there was any difference between the four samples in terms of mean self-reported VAS scores and mean self-reported EQ-5D utility indices on the basis of the UK tariff [15].

The TTO valuations derived for the four groups of health states were transformed using the same process used for the UK EQ-5D tariff [15] to ensure all health state values are bound between −1 and +1. The mean transformed TTO values and SDs are reported. For each variant, the effect of including the additional item was assessed by comparing the mean values with and without the additional item using paired t tests by the three core EQ-5D states. A level of 0.05 was assumed for statistical significance. Also, the mean values of EQ-5D states from the present study were compared with those obtained from the MVH study.

Regression analyses were used to further examine whether the impact of the bolt-on dimension on EQ-5D health state values differs by bolt-on variants and EQ-5D health states before and after controlling for sociodemographic characteristics. The general model is as follows:

$$y_{\mathrm{ij}}=\left(\alpha +\beta x_{\mathrm{ij}}+\delta q_{j}+{\theta r}_{j}+\gamma z_{i}\right)+\varepsilon_{\mathrm{ij}}$$

where *y*_*ij*_ are TTO utility values for health state *j* valued by respondent *i*; *i* = 1, 2, …, *m* represents individual respondents; *j* = 1, 2, …, *n* represents health states valued; ***x*** is the vector of dummy variables for the three EQ-5D core health states; ***q*** is the vector of dummy variables for each variant (including the EQ-5D and three bolt-on dimensions); ***γ*** is the vector of dummy variables for the three severity levels of the bolt-on dimensions; ***z*** is the vector of sociodemographic characteristics, including respondent’s sex, age, and experience of the condition of bolt-on dimensions; and *ε*_*ij*_ is an error term whose autocorrelation structure and distributional properties depend on the assumptions underlying the particular regression model used.

The dependent variable consisted of TTO values elicited from the four groups of respondents who valued EQ-5D states with and without a bolt-on dimension. Sets of dummy variables were used to represent the EQ-5D health states, the bolt-on variant, and the severity of the bolt-on dimension. Therefore, the model included as explanatory variables 1) two dummy variables (***x***) for the three core EQ-5D states (11121, 22222, and 22233), with state 11121 as reference; 2) three dummy variables (***q***) indicating the four variants of the questionnaire, with the EQ-5D (without a bolt-on dimension) used as the reference; 3) two dummy variables (***γ***) indicating the three severity levels of the bolt-on items, with level 1 used as the reference value; and 4) dummy variables (***z***) for various sociodemographic characteristics.

Because multiple valuations have been given by the same respondent and health state values may be clustered by respondents, the one-way error components random effects models were used to take account of the clustering of data by respondents and to allow for the fact that the error term may not be independent of the respondent.

SPSS 18 (SPSS, Inc., an IBM Company, Chicago, IL) was used for the descriptive statistical analysis. STATA version 10 (StataCorp, College Station, TX) was used for all regression analysis.

## Results

### Sample Sociodemographic Characteristics

The valuation survey was undertaken between March and June 2011. A total of 300 members of the public were successfully interviewed (75 for each variant) and their data included in the analysis.

The personal sociodemographic characteristics and the self-reported health status are reported in Table 1. The results of the chi-square test and analysis of variance suggested that there were no statistically significant differences between the four groups across most characteristics. The two exceptions were that people valuing the vision bolt-on reported fewer vision problems (*P* = 0.04) and the no bolt-on and tiredness bolt-on groups reported more experience in caring for others (*P* < 0.01). It is worth noting that tiredness was a much common health problem because there was a relatively high proportion of people reporting being extreme tired, which was not found for other dimensions. Also, the proportions of males and females across the samples were quite different, even though not statistically significant.

**Table 1:** Socioeconomic characteristics of respondents in the four groups to value health states of the EQ-5D and the EQ-5D with bolt-on dimensions.

**Characteristic**	**EQ-5D (n = 75)**	**EQ-5D + Hearing (n = 75)**	**EQ-5D + Vision (n = 75)**	**EQ-5D + Tiredness (n = 75)**
Age (y) (%)				
18–24	5	17	9	11
25–34	21	7	11	17
35–44	20	16	24	8
45–54	16	19	27	23
55–64	20	19	12	23
≥65	17	23	17	19
Sex: male (%)	32	40	49	39
Relationship status (%)				
Single	21	32	23	28
Married	53	40	60	48
Separated	3	7	6	5
Divorced	12	15	5	9
Widowed	11	5	5	9
Experience of serious illness (%)				
In yourself	29	33	23	37
In your family	68	68	71	79
In caring for others*	55	36	40	52
Main activity (%)				
Employment	52	36	45	39
Retired	24	29	27	35
Housework	6	12	9	6
Student	3	5	5	6
Seeking work	6	12	6	3
Other	8	5	6	11
Education after minimum school-leaving age (%)				
Yes	64	60	56	55
Degree: yes	29	27	29	25
Home ownership (%)				
Own home	71	65	75	69
Rent (local authority)	17	16	19	17
Rent (private sector)	12	19	7	12
Self-reported VAS score, mean ± SD	77.1 ± 21.2	80.9 ± 17.2	78.9 ± 17.8	74.7 ± 21.5
Self-reported EQ-5D index, mean ± SD	0.83 ± 0.26	0.80 ± 0.28	0.84 ± 0.28	0.75 ± 0.32
Self- reported health states (based on responses to the EQ-5D and the three bolt-on dimensions)				
Mobility				
Level 1	62	59	58	48
Level 2	13	15	17	27
Level 3	0	1	0	0
Self-care				
Level 1	70	70	66	67
Level 2	5	4	8	8
Level 3	0	1	1	0
Usual activities				
Level 1	62	61	61	52
Level 2	11	11	11	21
Level 3	1	2	3	2
Pain/discomfort				
Level 1	46	48	53	41
Level 2	24	22	18	27
Level 3	5	5	4	7
Anxiety/depression				
Level 1	57	58	63	57
Level 2	15	13	11	12
Level 3	3	4	1	6
Hearing				
Level 1	64	63	61	63
Level 2	11	11	14	11
Level 3	0	1	0	1
Vision*				
Level 1	44	43	61	45
Level 2	30	30	13	29
Level 3	1	2	1	1
Tiredness				
Level 1	39	40	42	40
Level 2	28	29	31	25
Level 3	8	6	2	10

EQ-5D, EuroQol five-dimensional questionnaire; VAS, visual analogue scale.

⁎ *P* < 0.05.

### TTO Health State Values

A total of 2697 TTO values were elicited from the 300 respondents. On average, each state was valued around 75 times. Summary statistics for the TTO values given to the nine EQ-5D health states (without bolt-on) and comparison with the MVH study [15] are given in Table 2. The EQ-5D values obtained in the present study were consistently higher than those obtained in the MVH study [15].

**Table 2:** Mean TTO values for all EQ-5D heath states in comparison to those in the MVH study.

State	Values from the current valuation study						Values from the MVH study
	N	Mean ± SD		Median	Min	Max	Mean
11121	76	0.94 ± 0.11		1.00	0.50	1	0.85
11112	75	0.93 ± 0.14		1.00	0.40	1	0.83
11122	75	0.87 ± 0.19		1.00	0.20	1	0.72
22222	74	0.71 ± 0.30		0.80	−0.30	1	0.50
22233	74	0.41 ± 0.40		0.43	−0.80	1	−0.14
21232	76	0.52 ± 0.40		0.50	−0.80	1	0.06
22323	75	0.46 ± 0.43		0.50	−0.93	1	0.04
33232	74	0.11 ± 0.40		0.01	−0.93	1	−0.33
33333	75	−0.02 ± 0.40		0.00	−0.93	1	−0.54

EQ-5D, EuroQol five-dimensional questionnaire; MVH, Measurement and Valuation of Health; TTO, time trade-off.

The descriptive statistics for TTO values given to the three core EQ-5D states and EQ-5D plus bolt-on states are summarized in Table 3. Mean values of the three core EQ-5D states were 0.41 (state 22233), 0.71 (state 22222), and 0.94 (state 11121), with an order that was consistent with severity of the states. The same ordering was maintained after adding the bolt-on items.

**Table 3:** Comparison between mean TTO values for the EQ-5D and the EQ-5D with add-ons.

EQ-5D		EQ-5D + Hearing			EQ-5D + Vision		EQ-5D + Tiredness	
**State**	**Mean ± SD**	**State**	**Mean ± SD**	***P***	**Mean ± SD**	***P***	**Mean ± SD**	***P***
11121	0.94 ± 0.11	111211	0.94 ± 0.13	0.89	0.94 ± 0.11	0.82	0.94 ± 0.14	0.71
		111212	0.90 ± 0.18	0.07	0.90 ± 0.13	0.01*	0.90 ± 0.15	0.06
		111213	0.85 ± 0.24	<0.01*	0.69 ± 0.28	<0.01*	0.82 ± 0.26	<0.01*
22222	0.71 ± 0.30	222221	0.80 ± 0.25	0.04*	0.74 ± 0.23	0.54	0.79 ± 0.26	0.09
		222222	0.77 ± 0.27	0.18	0.76 ± 0.21	0.25	0.74 ± 0.30	0.54
		222223	0.70 ± 0.30	0.82	0.59 ± 0.29	0.02*	0.72 ± 0.27	0.85
22233	0.41 ± 0.40	222331	0.40 ± 0.44	0.92	0.41 ± 0.35	0.99	0.45 ± 0.43	0.51
		222332	0.45 ± 0.44	0.56	0.41 ± 0.34	0.99	0.45 ± 0.42	0.52
		222333	0.36 ± 0.41	0.43	0.32 ± 0.33	0.16	0.34 ± 0.45	0.33

*Note. P* values are statistical result from the *t* test between EQ-5D health state values with and without the bolt-on item.

⁎ *P* < 0.05.

The EQ-5D values obtained in the present study were consistently higher than those obtained in the MVH study [15].

### Comparison of TTO Values of the EQ-5D with and Without Bolt-Ons

The results of *t* tests comparing TTO values between the three core EQ-5D states and the corresponding nine states with specific bolt-on dimensions are reported in Table 3. In most cases, the addition of level 1 (no problems) resulted in no change or increase in values; the addition of level 2 (moderate problems) had a mixed impact of decreasing and increasing the values; and the addition of level 3 (severe problems) reduced the values. This varied, however, between core EQ-5D states and variants of bolt-on.

For the mild EQ-5D state (11121), adding a level 1 bolt-on to it to form state 111211 did not change the mean value, and the mean values were identical across all variants. Adding on a level 2 to it to form state 111212 resulted in lower values for all three bolt-on dimensions, and the difference was statistically significant for vision and near significant for hearing (*P* = 0.07) and tiredness (*P* = 0.06). Adding on a level 3 to form state 111213 resulted in significantly lower mean health state values across all bolt-ons. Among the three bolt-ons, adding on a level 3 for vision showed the greatest impact on the TTO value: the mean value decreased from 0.94 to 0.69 compared with 0.85 for hearing and 0.82 for tiredness. Within each bolt-on, the TTO values were consistent with the severity of the states.

The pattern of values for the bolt-on items to the EQ-5D moderate (22222) and severe (22233) states was more complex. For state 22222, after adding level 1 or level 2 of the three bolt-on variants, the mean TTO values all increased but only level 1 hearing had a statistically significant effect. There was little impact of adding a level 3 for hearing and tiredness but a significantly lower value for the level 3 vision bolt-on.

For the severe state 22233, none of the bolt-on items had a statistically significant impact on the TTO value. After adding level 1 or level 2 of the bolt-ons, the mean TTO values showed no difference for vision, small increases for tiredness, and a slight increase for level 2 hearing but none of the differences was statistically significant. Although not statistically significant, the addition of level 3 led to a reduction in mean TTO values for all bolt-on variants.

### Regression Analysis

Table 4 presents the results of the regression analysis examining whether the impact of the bolt-on dimension on EQ-5D health state values differs by bolt-on and EQ-5D health states before (model 1) and after (model 2) controlling for sociodemographic characteristics. In model 1, the coefficients of the core health state dummy variables are statistically significant and are consistent with the severity of health states from mild, moderate, to severe. None of the bolt-on variants dummies is statistically significant, which demonstrates that there are no significant differences between the bolt-ons after controlling for sociodemographic characteristics. The values for the level 2 bolt-on (−0.015) were not significantly different from those for level 1, but there was a significant difference for the addition of level 3 (−0.113). Note that the level 0 dummy (EQ-5D states without bolt-on) was dropped because of collinearity with the bolt-on variant. After introducing sociodemographic variables, model 2 did not change the main trend of coefficients of core EQ-5D states, bolt-on variants, and levels in model 1. Coefficients of most sociodemographic characteristics were not statistically significant; in particular, experience of vision problems or caring for others did not significantly affect the health state valuations. There were two exceptions of marital status and main activities. Compared with people who are single, people who are married, divorced, or separated gave statistically significant higher values for the health states. There were no significant differences between health states valued by single and separated people. Compared with employed people, people who are seeking work gave statistically higher values.

**Table 4:** Random effect models for health state values.

**Health state**	**Model 1**	**Model 2 (n = 2219)**
Core states		
11121	Reference	Reference
22222	−0.151*	−0.151*
22233	−0.483*	−0.487*
Bolt-ons		
No bolt-on	Reference	Reference
Hearing	0.036	0.054
Vision	−0.009	0.003
Tiredness	−0.029	0.040
Bolt-on levels		
Level 1	Reference	Reference
Level 2	−0.015	−0.015
Level 3	−0.113*	−0.114*
Sex		
Male		Reference
Female		−0.019
Age (y)		
18–24		Reference
25–34		0.052
35–44		0.038
45–54		0.004
55–64		0.040
≥65		0.067
Marriage status		
Single		Reference
Married		0.091†
Separated		0.052
Divorced		0.094‡
Widowed		0.153†
None experience of serious illness		
In yourself		0.000
In your family		−0.028
In caring for others		−0.047
Main activities		
Employed		Reference
Retired		−0.036
Housework		0.013
Student		0.020
Seeking work		0.103†
Others		0.060
No education after minimum school-leaving age		0.057†
House ownership		
Own home		Reference
Rent from local authority		0.027
Rent from private sector		0.032
Self-reported health		
Hearing 1		Baseline
Hearing 2		−0.034
Hearing 3		0.114
Vision 1		Baseline
Vision 2		0.012
Vision 3		−0.137
Tiredness 1		Baseline
Tiredness 2		0.015
Tiredness 3		−0.065
Constant	0.903*	0.804*

⁎ *P* < 0.01.

† *P* < 0.05.

‡ *P* < 0.1.

## Discussion and Conclusions

Each of the bolt-on dimensions had a significant impact on values for EQ-5D health states. The extent and direction of the impact of the bolt-on varied according to the level of severity of the bolt-on dimension and the severity of the state to which it was added. In most cases, including a level 1 bolt-on resulted in no difference or higher values, the addition of level 2 was mixed, and the addition of level 3 led to lower values. For the mild EQ-5D state (11121), adding a level 1 bolt-on did not change the mean value; adding on a level 2 or level 3 resulted in lower mean health state values, and these differences were significant for level 2 vision and all level 3 bolt-on dimensions. The patterns for the addition of bolt-on items to the moderate and severe EQ-5D states were more complex. Adding a level 1 bolt-on to the moderate state led to higher values, but this was statistically significant only for the hearing bolt-on. The addition of a level 3 bolt-on to the moderate state led to statistically significantly lower values for the vision bolt-on but no difference for the hearing and tiredness bolt-ons. Adding a level 3 bolt-on to the severe state led to lower values; however, the difference was not statistically significant. There did not appear to be substantial differences between the three bolt-on variants, although the impact appeared to be marginally strongest for the vision bolt-on.

Results from the regression analysis showed that after adjusting for possible differences in sociodemographic characteristics between the groups, there are no significant differences in health state values between the three bolt-on variants. In terms of the impact of bolt-on dimensions on EQ-5D values, however, the results confirmed that that the impact does vary depending on the severity of the EQ-5D health state and the severity levels of bolt-on dimensions.

The results from this study have important implications for the further development and valuation of bolt-on dimensions for the EQ-5D. Because the impact of bolt-on dimensions depends on the severity of the EQ-5D state, a simple decrement will not be able to fully reflect the relationship between the existing dimensions and bolt-on dimensions and therefore an additive model to incorporate the bolt-on is likely to be inadequate. Although the results of this study cast doubt on the assumption that an additive model, rather than a multiplicative model, is the most appropriate model to value EQ-5D states with a bolt-on dimension, the current UK EQ-5D values set is not entirely additive through the inclusion of the N3 terms to allow for interactions. In a similar study that added a generic bolt-on dimension to a condition-specific preference-based measure, similar results were found that the impact on health state utility values was not simply additive [20]. Further research including the valuation of a larger number of health states using an orthogonal design is required to establish the impact of the bolt-on item on the values derived for the five EQ-5D dimensions and whether it is necessary to use more complex models (rather than the additive one) to incorporate possible interactions between the severity of the EQ-5D health states and the bolt-on dimensions or whether full valuations of the bolt-ons alongside the EQ-5D are required. This research suggests that all three potential bolt-on dimensions could benefit from further examination in this way.

Our results differ from those of an earlier study that investigated the impact of including “tiredness” as a dimension within the EQ-5D (i.e., a potential EQ-6D) using the VAS [12]. We found that the inclusion of a level of “no tiredness” on the bolt-on led to higher values compared with no bolt-on, as well as lower values reflecting “extreme tiredness.” One could hypothesize that differences between the two studies could be due to the combinations of levels each has chosen to investigate. This appears not to be the case, however, because both studies included a common health state (11121). The study by Gudex found that the addition of level 2 tiredness to state 11121 did not significantly affect the mean value, whereas our study found that it was associated with a near significantly lower value. There are notable differences between the two studies that could perhaps explain the discrepancy, including the valuation methods and the number of levels/labeling of the tiredness dimensions. Gudex used the VAS, whereas this study used TTO. In addition, the tiredness bolt-on dimension consisted of two possible levels in the study by Gudex, whereas the bolt-on dimension in this study included three levels. However, results similar to those of this study were reported in a previous study that added a sleep dimension to the EQ-5D [11]. A significantly higher mean value was found after adding level 1 (“I have no problems with sleep”) to a moderate EQ-5D state (11233), but no statistically significant differences were found when various severity levels of the sleep dimension were added to other five relatively moderate or severe EQ-5D states.

The EQ-5D values obtained in the present study were consistently higher than those obtained in the MVH study [15]. This is consistent with some international valuation studies of EQ-5D health states conducted since the MVH study, which have also reported higher mean TTO values compared with those reported by the original MVH study. This justifies the current within-study comparison design *[21]*.

One limitation of the study is that the interviews were based in a specific area of the United Kingdom and may not be generalizable to other countries or indeed areas of the United Kingdom although there is no clear reason to assume that the pattern of results would be different elsewhere. Overall the four groups valuing each of the bolt-on and the EQ-5D were well balanced; however, there were differences based on two background questions: namely, experience of caring for others and self-reported vision problems. The results of the regression analysis show that these characteristics did not have a significant impact on values and are therefore unlikely to affect comparisons between the groups. Although the development of bolt-on dimensions came from a brief review of existing measures and the labels within the dimensions built on the framework of the EQ-5D, another limitation of the study is that the development process for the bolt-on dimensions did not include qualitative research and psychometric testing for this stage of the research. This is because the primary focus of this exploratory study was to establish whether the proposed bolt-on dimensions have an impact on EQ-5D values, and if so, to further develop methods for valuing the bolt-on dimensions and incorporating them with EQ-5D values. It will be important to examine the psychometric properties and acceptability to respondents before routine use of the bolt-ons in future studies.

The primary aim of the research was to examine the methodology related to the valuation of bolt-on measures and how the bolt-on dimensions may affect EQ-5D health state values. We considered using the five-level EQ-5D (EQ-5D-5L) to test this methodological issue, but chose the three-level EQ-5D (EQ-5D-3L) for several reasons. First, the number of health state valuations required, and therefore sample size of respondents, would have to be much larger for the investigation of this methodological issue using the EQ-5D-5L. Second, the identification of our bolt-on dimensions was built on systematic reviews of the EQ-5D-3L’s performance in hearing, vision impairment, and cancer. At the time, there was little empirical evidence on the performance of the newly developed EQ-5D-5L in these conditions of interests. Third, the new valuation protocol of the EQ-5D-5L was under development and was not available when we started, and so we used the EQ-5D-3L and a modified MVH protocol. This study has focused on the three-level version (EQ-5D-3L) for the EQ-5D; therefore, future research is needed to test whether similar results would be seen with the five-level version (EQ-5D-5L) [9].

Given the design, it is inevitable that the bolt-on dimension varied more from the core dimensions for some of the health states. When a level 1 bolt-on dimension was added to the severe core state and when the level 3 bolt-on dimension was added to the mild core state, it may have made the bolt-on dimension “stand out” more. As demonstrated in Table 4, however, the evidence is mixed because state 111213 for all three bolt-on variants was significantly different from the EQ-5D state 11121 and the only two significantly different states from 22222 were 222221 for hearing and 222223 for vision; however, none of state 222331 for the three bolt-on variants was significantly different from 22233 as expected.

During the valuation task, short versions of the health state cards were presented to respondents. These cards did not contain additional information in parentheses for bolt-on dimensions (e.g., use of aids glasses and hearing aids) and usual activities (e.g., work, study, housework, family, or leisure activities). The additional information, however, was presented when respondents were asked to fill in the questionnaires as part of their self-rated health. The interviewer’s debriefing showed that no respondents asked questions about the use of equipment while completing the valuation with health state cards. It remains unclear whether the respondents take this into consideration when giving their values.

A key feature of the EQ-5D is that it can be used across a range of conditions or diseases. This has a substantial advantage for economic evaluation and health care decision making because it means that decisions can be based on a common measure and applied consistently across evaluations. The potential development of EQ-5D bolt-on items could facilitate greater sensitivity in the measurement of HRQOL for specific conditions. It could also lead, however, to some variations in measurement between conditions and detract from the advantages of using a generic instrument. Including the EQ-5D as the basis for measurement and following a common valuation methodology may reduce the potential for inconsistencies in the valuations obtained. The results of this research suggest that simple valuation of these bolt-on items may not be possible; however, further research is required to confirm this exploratory finding.

This exploratory study examined and compared the effect of including the three bolt-on dimensions on the EQ-5D health states. For future bolt-on studies, we recommend (1) conducting a valuation of a larger number of health states selected on the basis of statistical theory (e.g., the orthogonal design) to understand the impact of the additional dimensions and the development of a value algorithm; (2) exploring more complex models (rather than the additive one) to incorporate the severity of the EQ-5D health states and the impact of the bolt-on dimensions. The design of valuation studies should reflect this issue. We also recommend that the standard process be fully applied to develop and test psychometric performance of the bolt-on dimensions at a certain stage. We note, however, that bolt-ons need to have an impact on valuations and have good psychometric properties, and the order in which the two criteria are examined depends on the focus of the study and is indeed a matter of which is most efficient. This study was designed to be the first stage of the valuation research to assess the impact, including the direction of impact, of bolt-on dimensions on health states covering a range of severity. The second stage of the research would be to undertake a full valuation study using an orthogonal design that will include more health states and a larger sample of respondents, and to facilitate an estimate of the value algorithm for bolt-on measures.

## References

[1] Brazier J.E., Fitzpatrick R. (2002). Measures of health-related quality of life in an imperfect world: a comment on Dowie. Health Econ.

[2] Brazier J.E., Rowen D., Mavranezouli I. (2012). Developing and testing methods for deriving preference-based measures of health from condition-specific measures (and other patient-based measures of outcome). Health Technol Assess.

[3] Brooks R. (1996). EuroQol: the current state of play. Health Policy.

[4] National Institute for Health and Care Excellence (2008).

[5] National Institute for Health and Care Excellence (2013).

[6] Tosh J., Brazier J., Evans P., Longworth L. (2012). A review of generic preference-based measures of health-related quality of life in visual disorders. Value Health.

[7] Papaioannou D., Brazier J., Parry G. (2011). How valid and responsive are generic health status measures, such as EQ-5D and SF-36, in schizophrenia? A systematic review. Value Health.

[8] Brazier J., Connell J., Papaioannou D. (2014). A systemetic review, psychometric analysis and qualitative assessment of generic preference-based measures of health in mental health populations and the estimation of mapping functions from widely used specific measures. Health Technol Assess.

[9] Herdman M., Gudex C., Lloyd A. (2011). Development and preliminary testing of the new five-level version of EQ-5D (EQ-5D-5L). Qual Life Res.

[10] Krabbe P.F., Stouthard M.E., Essink-Bot M.L., Bonsel G.J. (1999). The effect of adding a cognitive dimension to the EuroQol multiattribute health-status classification system. J Clin Epidemiol.

[11] Yang Y., Brazier J., Tsuchiya A. (2014). Effect of adding a sleep dimension to the EQ-5D descriptive system: a “bolt-on” experiment. Med Decis Making.

[12] Gudex C. Are we lacking a dimension of energy in the EuroQol instrument? Paper presented at: the 8th Pleanary Meeting of the EuroQol Group, Lund, Sweden, 1991.

[13] Yang Y., Longworth L., Brazier J. (2013). An assessment of validity and responsiveness of generic measures of health-related quality of life in hearing impairment. Qual Life Res.

[14] Wailoo A, Davis S, Tosh J. The Incorporation of Health Benefits in Cost-Utility Analysis Using the EQ-5D: A Report by the NICE DSU. 2010. Available from: http://www.nicedsu.org.uk/. [Accessed November 5, 2014].

[15] Dolan P. (1997). Modeling valuations for EuroQol health states. Med Care.

[16] Dolan P., Gudex C., Kind P., Williams A. (1996). The time trade-off method: results from a general population study. Health Econ.

[17] Department of Health. Health Survey for England. 2006. Available from: http://wwwicnhsuk/pubs/hse06cvdandriskfactors). 2006. [Accessed September 18, 2010].

[18] AFD. Names and Numbers software 2012. Available from: www.afd.co.uk. [Accessed January 15, 2011].

[19] C G (1994).

[20] Brazier J., Rowen D., Tsuchiya A. (2011). The impact of adding an extra dimension to a preference-based measure. Soc Sci Med.

[21] Tsuchiya A., Brazier J., Roberts J. (2006). Comparison of valuation methods used to generate the EQ-5D and the SF-6D value sets. J Health Econ.

